# Indonesian National Growth Reference Charts Better Reflect Height and Weight of Children in West Java, Indonesia, than WHO Child Growth Standards

**DOI:** 10.4274/jcrpe.galenos.2020.2020.0044

**Published:** 2020-11-25

**Authors:** Novina Novina, Michael Hermanussen, Christiane Scheffler, Aman B. Pulungan, Yoyos Dias Ismiarto, Yudhie Andriyana, Vitriana Biben, Budi Setiabudiawan

**Affiliations:** 1 Universitas Padjadjaran Faculty of Medicine, Department of Child Health, Bandung, Indonesia; 2 Aschauhof, Eckernfoerde-Altenhof, Germany; 3 University of Potsdam, Human Biology, Potsdam, Germany; 4 Universitas Indonesia-Ciptomangunkusumo Hospital, Faculty of Medicine, Department of Child Health, Jakarta, Indonesia; 5 Universitas Padjadjaran Faculty of Medicine, Department of Orthopedics and Traumatology, Bandung, Indonesia; 6 Universitas Padjadjaran Faculty of Mathematics and Natural Sciences, Department of Statistics, Bandung, Indonesia; 7 Universitas Padjadjaran Faculty of Medicine, Department of Physical Medicine and Rehabilitation, Bandung, Indonesia

**Keywords:** Anthropometric measurement, Indonesian National Growth Reference Charts, World Health Organization Child Growth Standards, Bandung District children, undernutrition

## Abstract

**Objective:**

The Indonesia Basic Health Research 2018 indicates that Indonesian children are still among the shortest in the world. When referred to World Health Organization Child Growth Standards (WHOCGS), the prevalence of stunting reaches up to 43% in several Indonesian districts. Indonesian National Growth Reference Charts (INGRC) were established in order to better distinguish between healthy short children and children with growth disorders. We analyzed height and weight measurements of healthy Indonesian children using INGRC and WHOCGS.

**Methods:**

6972 boys and 5800 girls (n=12,772), aged 0-59 months old, from Bandung District were measured. Z-scores of length/height and body mass index were calculated based on INGRC and WHOCGS.

**Results:**

Under 5-year-old Indonesian children raised in Bandung are short and slim. Mean height z-scores of boys is -2.03 [standard deviation (SD) 1.31], mean height z-scores of girls is -2.03 (SD 1.31) when referred to WHOCGS indicating that over 50% of these children are stunted. Bandung children are heterogeneous, with substantial subpopulations of tall children. Depending on the growth reference used, between 9% and 15% of them are wasted. Wasted children are on average half a SD taller than their peers.

**Conclusion:**

WHOCGS seriously overestimates the true prevalence of undernutrition in Indonesian children. The present investigation fails to support evidence of undernutrition at a prevalence similar to the over 50% prevalence of stunting (WHOCGS) versus 13.3% (INGRC). We suggest refraining from using WHOCGS, and instead applying INGRC that closely mirror height and weight increments in Bandung children. INGRC appear superior for practical and clinical purposes, such as detecting growth and developmental disorders.

**What is already known on this topic?**

World Health Organization Child Growth Standards (WHOCGS) are used worldwide to interpret anthropometric measurement in children, except for those countries who have their own growth charts. In 2019, Indonesian National Growth Reference Charts (INGRC) were created, based on data from the Indonesia Basic Health Research 2013.

**What this study adds?**

Growth of Indonesian children is not well represented by WHOCGS, as these standards overestimate the true prevalence of undernutrition. INGRC should be used for practical and clinical purposes.

## Introduction

Stunting is considered one of the most prevalent health problems in Indonesia. Stunting is defined as the percentage of children whose height-for-age is below minus two standard deviations (SD) (moderate stunting) or minus three standard deviations (severe stunting) from the median of the World Health Organization Child Growth Standards (WHOCGS). Stunting is the impaired growth and development that children experience from poor nutrition, repeated infection, and inadequate psychosocial stimulation. The term “stunting” is commonly used to indicate chronic mal- or undernutrition during critical periods of growth and development, especially during the first 1000 days of life (1).

The Indonesia Basic Health Research 2018 indicated that Indonesian children are still among the shortest in the world. When referred to WHOCGS, the prevalence of stunting reaches as much as 43% in several Indonesian districts (2).

In 2015, Indonesia, along with other countries in United Nations, agreed on Sustainable Development Goals (SDGs) to be achieved by 2030 in order to reduce poverty, lessen the wealth gap, and protect the environment. SDGs consist of 17 core goals and 169 targets. The second goal is “to end hunger, achieve food security and improved nutrition and promote sustainable agriculture” (SDGs 2015). Stunting, since it is said to be related to nutritional status, was put on the SDGs second goal’s indicator framework: to eradicate all forms of malnutrition including achieving the 2025 target on stunting and wasting, and improve nutritional needs (3,4). The WHO’s Nutrition Landscape Information System defines stunting as length for age (LAZ) or height for age (HAZ) <-2 SD, and wasting as BMI for age (BAZ) <-2 SD from the median of the WHO Child Growth Standards (5). The Indonesia Basic Health Research 2018 showed that the number of stunted children is still high. While stunting is related to many factors, such as infections or psychosocial neglect, it is also associated with nutritional intake. The Indonesian government has made plans to improve the nutritional intake of Indonesian children by issuing Presidential Decree No. 42 of 2016 about the national movement for the Acceleration of Nutrition Improvement with a focus on the first 1000 days of life which prioritizes joint efforts between government and the community through coordinating stakeholder’s participation and awareness towards accelerate community nutrition improvement (6).

This concept has been questioned. Stunting *per se* is not a synonym of malnutrition (7). A recent study performed in elementary school children from three Indonesian provinces, focused on the relationship between nutritional status and height and was unable to present evidence that stunting resulted from undernutrition in these children (7). This view is supported by data obtained in the Indonesia Basic Health Research 2018 (Table 1). The data illustrate the discrepancy between the large number of stunted and the comparably low number of wasted children, and question that stunting reflects undernutrition (2).

Biological and socio-economic factors are known to influence child growth. The great variety of clinical conditions associated with short stature further complicates identifying reasons for poor growth. Accurate and regular anthropometric measurements are essential, easy and inexpensive tools to help disentangling the complicated regulation of growth and to detect relevant growth and development disorders (8). As clinical practice has shown that WHOCGS seem to provide little help in this intricate matter, many countries have meanwhile constructed national growth reference charts (9,10,11,12).

Indonesia is an archipelago country formed by 17,508 islands. Its population ranks at number four in the world. There are five main islands in Indonesia: Sumatra, Java, Kalimantan, Sulawesi, and Papua (13) housing an extremely heterogeneous composition of ethnically, culturally and economically very different populations. In 2019, National Growth Reference Charts for Indonesian (INGRC) children were established, based on the Indonesia Basic Health Research 2013. The samples were taken from all Indonesian provinces, and considered representative for the Indonesian child population (9). The present study was undertaken to test the reliability of the new INGRC. The aim of this study was to compare WHOCGS with the new INGRC in under 5-year-old Indonesian children, raised in the Bandung District area. As Indonesian national references are based on healthy Indonesian children, we expected INGRC to better fit Indonesian growth patterns than WHOCGS. Yet, as short stature is commonly associated with chronic undernutrition, we focused on body mass index (BMI) as a rough indicator of the nutritional status, its association with height, and particularly, on the shape of the height and the BMI distributions. Starving and malnourished populations are on average short, but a population is never equally affected by starvation or malnutrition. Some people may receive enough food, their children grow well or almost well, others may receive too little and their children stop growing and become stunted. Children may also differ in sensitivity to food deprivation: some may stop growing early, others may grow even when food rations are very poor. Unequal food distribution and unequal biological responses do not only affect mean values of height and BMI, they will affect height and BMI variation. Unequal living conditions will raise height and BMI variance.

Indonesian’s Global Hunger Index (GHI) is 20.1, which indicates that Indonesian children are considered “seriously” affected by starvation. GHI values are determined for four indicators: undernourishment (insufficient caloric intake), wasting among children under 5 years of age/low WHZ (weight-for-length) (acute undernutrition), stunting among children under 5 years of age/low HAZ (chronic undernutrition) and mortality rate of children under 5 years of age (results from undernutrition and unhealthy environment) (14). As Indonesian children, regardless of their nutritional state, are generally shorter and lighter than prescribed by WHOCGS, they will always be categorized as chronically undernourished as long as these growth charts are used.

We hypothesized that:

1) height and BMI of under 5-year-old Indonesian children raised in the Bandung District area would be smaller than suggested by WHO Child Growth Standards.

2) the variance of height and BMI would be broader than suggested by WHO Child Growth Standards.

In addition, we hypothesized:

3) that wasted children (BMI <-2 SD, using WHOCGS) are shortest.

## Methods

Length/height and weight of 12,772 healthy children, 6,972 (54.6%) boys and 5800 girls aged 0-59 months, from Bandung District area, were measured. The sample was taken from 31 sub-districts and included both urban and rural children from the whole spectrum of economic provenance, including children both from impoverished and affluent backgrounds. Length/height and weight measurements were performed according to a standard procedure (15). The weight was measured to the nearest 100 grams using Indonesian Dacin scale, which is the most commonly used scale for children in Indonesia Primary Maternal and Child Health Care. The length of children ≤2 years old was measured using an infantometer in a supine position. In children >2 years old, height was measured using microtoise stadiometer to the nearest millimeter (Dacin scale was manufactured by Sanes Sumber Makmur, both infantometer dan microtoise were manufactured by GEA Medical).

The data were obtained from the Health Office of Bandung District’s Nutritional Status Monitoring for Children Under 5 Years Old. Measurements were done in March 2019 by healthcare providers using standardized tools. Written consent for Nutritional Status Monitoring was obtained from parents according to the policy of Bandung District Health Office. Weight, height and BMI were compared to WHO Child Growth Standards and INGRC (9,16).

This study was approved by the Ethics Committee of Faculty of Medicine, Universitas Padjadjaran, Ethical Approval no 1170/UN6.KEP/EC/2019, and conformed to the ethical guidelines of the Declaration of Helsinki.

### Statistical Analysis

Statistical analyses were performed using SPSS, version 24.0 (IBM Inc., Armonk, NY, USA). All data were plotted on charts using The R project for statistical computing version 3.5.0 (17). The F-test was used to compare variances.

Children were anonymized and de-identified before analysis.

## Results

**Figure 1:**
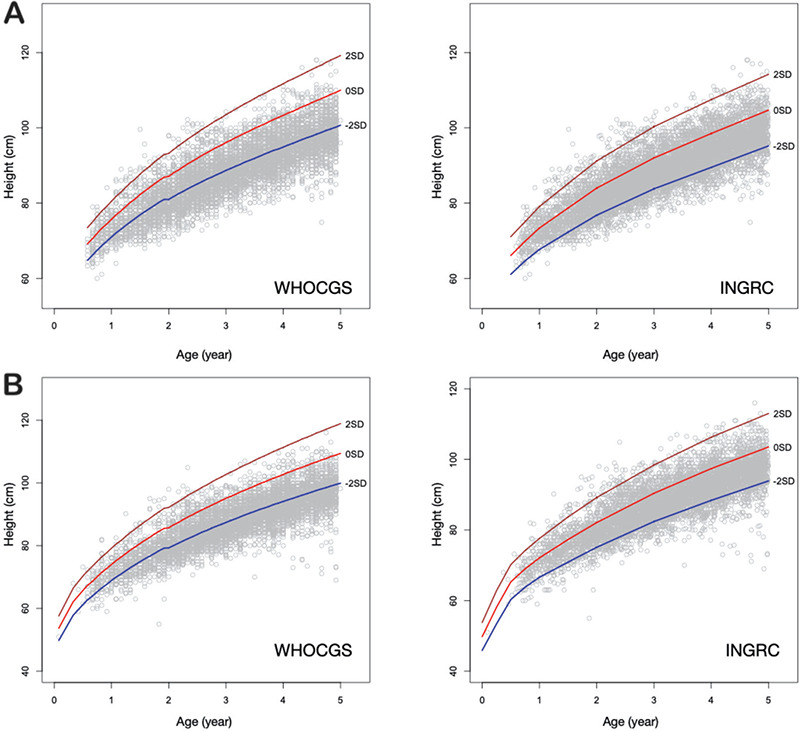
Height of Bandung District Boys (A) and Girls (B) plotted on WHOCGS and INGRC. The children from Bandung District are short. More than 50% must be considered stunted according to WHOCGS. WHOCGS: World Health Organization Child Growth Standards, INGRC: Indonesian National Growth Reference Charts

**Figure 2:**
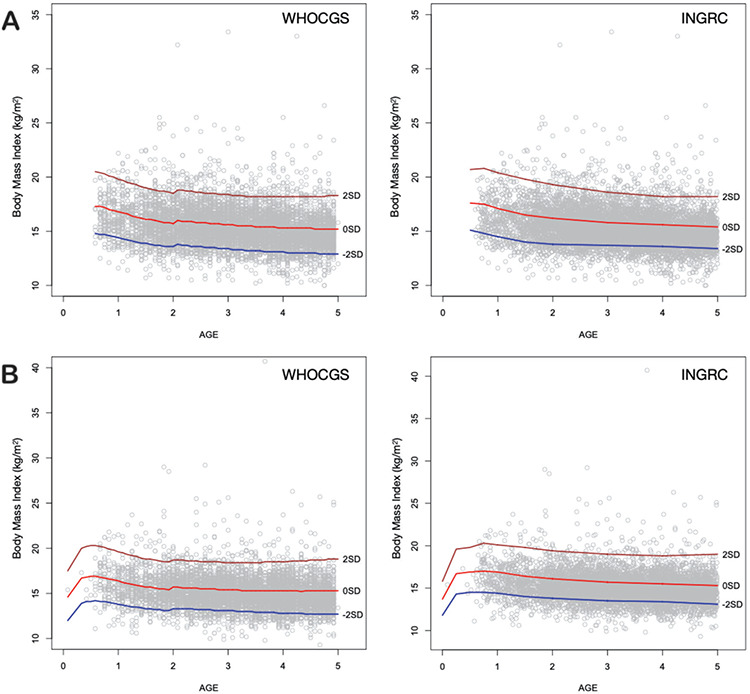
BMI of Bandung District Boys (A) and Girls (B) plotted on WHOCGS and INGRC. The number of obese children is very small. WHOCGS: World Health Organization Child Growth Standards, INGRC: Indonesian National Growth Reference Charts, BMI: body mass index

Under 5-year-old Indonesian children raised in the Bandung District area are short (Figure 1) and slim (Figure 2). Mean height z-scores of boys was -2.03 (SD 1.31), mean height z-scores of girls was -2.03 (SD 1.31) when referred to WHOCGS, indicating that more than 50% of these children are stunted. When referred to INGRC, the percentage of stunted children declined to 13.3%. Depending on the growth reference used, between 9% (WHOCGS) and 15% (INGRC) of the Bandung District area children are wasted (Table 2).

Table 2 illustrates to what extent the choice of the growth reference chart influences the apparent percentage of stunted and wasted children. Whereas INGRC identifies 10.8% moderately, and 2.5% severely stunted children in Bandung District, reference to WHOCGS suggested that 34.72% were moderately, and 21.59% were severely stunted.

**Figure 3:**
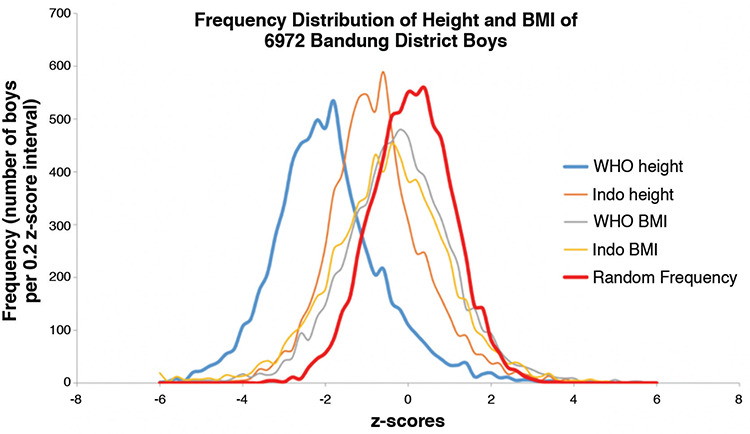
Frequency distribution of height and body mass index of 6,972 Bandung District boys WHO BMI: World Health Organization Child Growth Standards body mass index, Indo BMI: Indonesian National Growth Reference Charts body mass index

**Figure 4:**
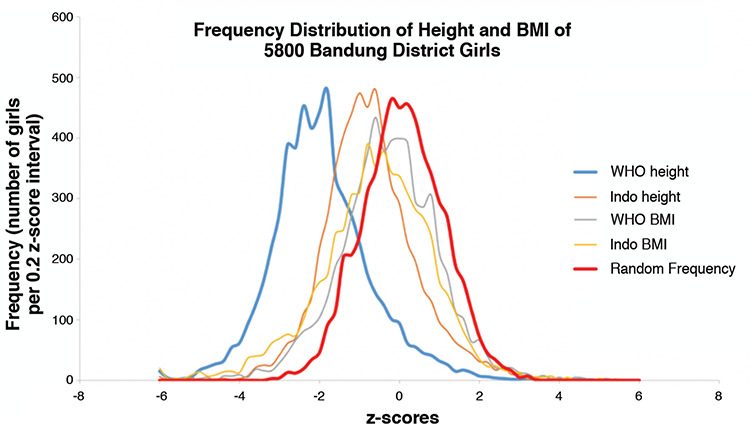
Frequency distribution of height and body mass index of 5,800 Bandung District girls WHO BMI: World Health Organization Child Growth Standards body mass index, Indo BMI: Indonesian National Growth Reference Charts body mass index

Figures 3 and 4 illustrate the frequency distributions of LAZ/HAZ and BAZ based on WHOCGS and on INGRC, and a virtual cohort with random z-scores defined by mean values of zero, and standard deviations of one. Neither LAZ/HAZ nor BAZ are normally distributed. Bandung children are shorter and slimmer than suggested by WHOCGS and by INGRC. True LAZ/HAZ and BAZ distributions are broader than the virtual random distributions, and the LAZ/HAZ distributions are significantly skewed (p<0.001). Table 3 and Figures 3 and 4 indicate that Bandung District children are heterogeneous, with substantial subpopulations of tall children as indicated by the elongated right leg of the height z-score curves.

**Figure 5:**
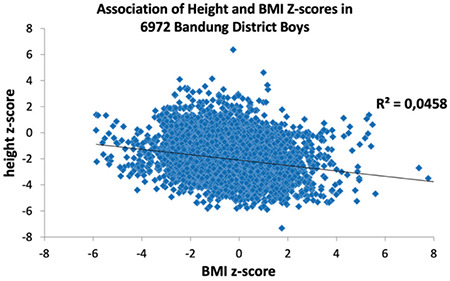
Association of height and body mass index z-scores in 6,972 Bandung District boys BMI: body mass index

**Figure 6:**
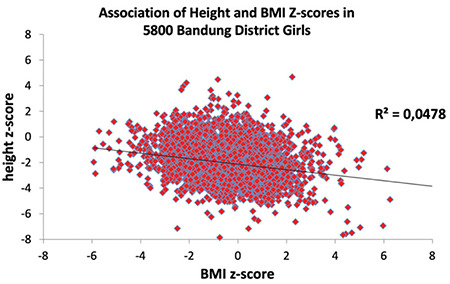
Association of height and body mass index z-scores in 5,800 Bandung District girls BMI: body mass index

The broadened and skewed distributions of height and weight z-scores suggest inequality among Bandung District children. In view of the common perception that short stature is considered an indicator of chronic undernutrition, and BMI an indicator of the nutritional status, we also studied the association between height and BMI (Figure 5, 6).

We found the opposite of what was expected, that is being thin is not associated with being short. The association between LAZ/HAZ and BAZ is negative. Slim children are taller. In order to further scrutinize this association, we investigated LAZ/HAZ of those children who are by definition considered wasted (BAZ <-2 SD, WHOCGS). LAZ/HAZ of wasted boys was -1.3 (SD 1.48), LAZ/HAZ of wasted girls was -1.34 (SD 1.44). Wasted Bandung District children are on average 0.7 SD taller than their peers (Table 3: all boys: -2.03 SD, all girls -2.05 SD; p<0.001 for both sexes).

## Discussion

The Indonesia Basic Health Research 2018 indicated that Indonesian children are short. This also applied to children raised in the Bandung District area. When referred to WHO Child Growth Standards (WHOCGS), more than 50% of these children are stunted. Under 5-year-old Bandung District children are also slim. This confirms our first hypothesis.

Bandung District children are heterogeneous. The variance of height and BMI is broader than suggested by WHOCGS, confirming our second hypothesis. The combination of being on average short and slim and the heterogeneity of the population of Bandung children might, at first view, support the general perception that these children suffer from malnutrition, and that length‐for‐age may indeed, serve as an appropriate indicator for chronic nutritional deficiency (18). However, this impression is deceptive. The present study illustrated that being slim is not associated with being short. The present analysis clearly rejects the third hypothesis. The very slim (wasted) children with BMI <-2 SD (WHOCGS) are not the shortest. On the contrary, children who are by definition “wasted” are on average 0.7 standard deviations taller than their peers. Stunting is not a synonym of malnutrition (7).

The observation that the thinnest children are tallest questions the current concept of nutrition-dependent growth regulation. An estimated 50 percent prevalence of stunting when using WHOCGS can by no means, plausibly suggest that half of the Bandung District children suffer from chronic undernutrition, repeated infections or child neglect and lack of psychosocial stimulation.

The 2019 Food Security and Vulnerability Atlas (FSVA) classified districts in Indonesia into one to six priority groups, from the most food-insecure to the most food-secure. FSVA exhibits Districts in West Java as priority 5 (22%) and 6 (78%) which indicating appropriate food-security (19). Studies in respect of availability of major food products, including fruits, vegetables, livestock and fisheries in West Java, especially Bandung District, revealed steady and secure food diversification policies, which are able to cover production, distribution, access and demand among the community (20,21). Based on Central Bureau of Statistics Republic of Indonesia, the Gross Regional Product Nominal (GRP Nominal) per capita of West Java Province on 2019 ranked the third highest of 34 provinces and the Gross Regional Domestic Revenue of Bandung District on 2016 ranked 27 out of 514 districts and cities in Indonesia. Bandung also ranked 114 of 120 in Global City Competitiveness Index in 2012 (22,23,24). Children in Bandung grew in supportive atmosphere. The Bandung city government provides comprehensive attention on children’s health, education and psychosocial needs. This leads Bandung won the Child-Friendly City award from the Ministry of Women’s Empowerment and Child Protection for the third time in a row on 2019 (25).

Bandung District is wealthy, with no evidence of food shortage or clinical signs of malnutrition in the children raised in this area. The claim that 50% of the healthy Bandung District children as mal- or undernourished, is unsubstantiated. The observation that the slimmest children of Bandung District grew best, further challenges the prevalent concept of length‐for‐age being the indicator of choice in monitoring chronic nutritional deficiency.

Yet, the question remains: why are these children short, and why do the slimmest children grow best? Modern nutrition studies do not throw light on this matter, but numerous historic observations match our findings.

Most Europeans of the 19th century were shorter than modern Indonesians. In spite of the tremendous wealth of the European nations at that time, European children grew poorly. Even upper-class urban adolescents grew less than modern Indonesians (26). This almost ubiquitous pattern of historic European growth changed after World War I. In 1919, the German pediatrician Schlesinger wrote: “In the second year of the war (World War I), there were more than a few groups of boys from the public citizens’ and advanced educational schools who were 1-2 cm taller than in the year 1913 (before the war). This difference in the second year of the war was even more conspicuous, as at the same time there was a very clear and not very small weight loss”. And based on measurements of the loss in body fat, both in absolute terms and related to body height, Schlesinger wrote in 1924 that: “even more regular is the deficit in weight in 1916 versus 1913, when taking into account the length of the body, which in this period has partly shifted in the opposite direction” (27). Even though the children became slimmer, they nevertheless grew taller. The rapid secular height trends after World War I coincided with the political transition from feudalism in the Imperial period to socialist or democratic structures. The adolescents raised at that time, anticipated rapid political changes, liberation and equal opportunities, and closely coinciding with the political changes, increased in height by one to two millimeters per annual cohort (28).

Human growth is regulated by biological factors such as nutrition, genetics, and general health (26), but recent evidence also suggests social, economic, political and emotional (SEPE) factors (29). Political transition appears to be one of the most distinguished promotors of human growth factors. Absence of political oppression is a growth stimulus. Hermanussen and Scheffler (28) (2016) discussed community effects on body height, and considered stature as a social signal. The data of the present investigation indirectly support this vision. Indonesia is still a developing country but it shows advancements in democratisation, personal freedom and equal opportunities. The recent political achievements provide possible explanations for the negative association between height and BMI. Bandung children are slim, but not because of nutrition deficits. The subpopulation of the very slim and taller than average children of Bandung, mirrors the SEPE situation that was prevalent in central Europe after World War I, at the dawn of political modernization (29).

Use of the WHOCGS categorizes more than 50% of the healthy Bandung District children as “stunted”, thereby alleging chronic mal-and undernutrition (1) of these children. The present investigation fails to support evidence for this concept. We suggest refraining from using global growth charts, and instead strongly support applying the new INGRC. These charts are based on data from Indonesia Basic Health Research 2013. They also closely mirror height and weight increases of Bandung children, and appear superior for practical and clinical purposes, such as detecting growth and developmental disorders.

In view of body height as a mirror of the SEPE situation of a country (29), we consider frequent updating the INGRC essential. We are convinced that coinciding with the political modernization, Indonesian children will in the near future follow the same global growth standards for height and weight as suggested by the WHO. Child health care and prevention require relevant national references for height, weight and BMI.

### Study Limitations

The study was performed in a cross-sectional sample of infants and children, with no detailed information on individual nutrition, individual health, individual repeated infection and individual socio-economic background. Thus, the data do not allow direct inferences between growth, nutritional situation, morbidities, psychosocial status, and socio-economic circumstances. Instead we used data of the Indonesia FSVA with verified local information. Considering the GRP Nominal per capita of West Java Province, the Gross Regional Domestic Revenue of Bandung District and Bandung children’s health, education and social services, it seems that Bandung District is in good economy condition, adequate psychosocial stimulation status, with good food security and absence of child poverty.

## Conclusion

The WHOCGS seriously overestimates the true prevalence of undernutrition in Indonesian children. The present investigation fails to support evidence of undernutrition. We suggest refraining from using WHOCGS, and instead applying INGRC. These latter charts closely mirror height and weight increments in Bandung children. They appear superior to currently used WHO Child Growth Standards for practical and clinical purposes, such as detecting growth and developmental disorders.

## Figures and Tables

**Table 1:**
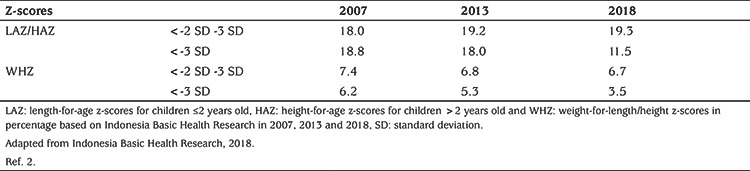
Percentage of Indonesian children under five years old below standard cut-offs for length and weight-for-height

**Table 2:**
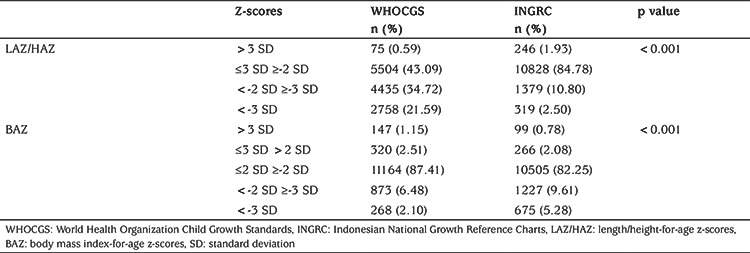
Number and percentage of severely stunted [length/height-for-age z-scores (LAZ/HAZ) <-3 SD], moderately stunted LAZ/HAZ <-2 SD, normal, and tall LAZ/HAZ > 3 standard deviation (SD) children; and severely wasted [body mass index for-age z-scores (BAZ) <-3 SD], moderately wasted BAZ <-2 SD, normal, overweight BAZ >2 SD, and obese children BAZ >3 SD. P values (Mann-Whitney U test) refer to the difference between World Health Organization Child Growth Standards and Indonesian National Growth Reference Chart

**Table 3:**
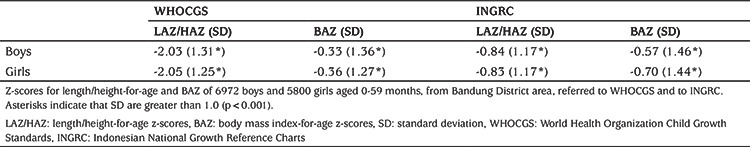
Mean z-scores for length/height-for-age and body mass index-for-age
